# The OCEANIC trial journey: from failure to breakthrough?

**DOI:** 10.1093/ehjcvp/pvag047

**Published:** 2026-06-27

**Authors:** Valeria Caso, Hugo ten Cate, Isabelle C van Gelder

**Affiliations:** Neurology-Stroke Unit, Saronno Hospital, ASST-Valle Olona, 21047 Saronno, Italy; Department of Internal Medicine, Thrombosis Expertise Center, Maastricht University Medical Center (MUMC+) CARIM—Cardiovascular Research Institute Maastricht, Maastricht University, PO Box 616, 6200 MD Maastricht, The Netherlands; Department of Cardiology, University Medical Center Groningen (UMCG), University of Groningen, 9713 GZ, Groningen, The Netherlands

## Introduction

Prevention of ischaemic stroke remains a major global health priority, affecting millions of individuals worldwide.^1^Antithrombotic strategies, however, differ fundamentally according to stroke mechanism. Primary prevention of cardioembolic stroke, most commonly in patients with atrial fibrillation (AF), relies on oral anticoagulation, whereas secondary prevention after non-cardioembolic ischaemic stroke is largely based on antiplatelet therapy. Despite substantial progress, residual ischaemic risk remains clinically significant, underscoring the need for more effective and safer therapeutic approaches.

## Asundexian in secondary stroke prevention: a recent success story

The OCEANIC-STROKE trial addresses a central unmet need in secondary stroke prevention: how to further reduce recurrent ischaemic events in patients with recent non-cardioembolic ischaemic stroke or high-risk transient ischaemic attack (TIA) without increasing the risk of major or intracranial bleeding.^2^ Although acute stroke care and antiplatelet-based secondary prevention have improved substantially, residual recurrence risk remains clinically relevant, especially in patients with atherosclerosis, multiple vascular risk factors, or prior cerebrovascular disease.^3^

Over recent years, secondary prevention after minor non-cardioembolic ischaemic stroke or high-risk TIA has moved towards early treatment intensification. Since 2021, international guidelines have recommended short-term dual antiplatelet therapy (DAPT), based on randomized evidence showing that aspirin–clopidogrel or aspirin–ticagrelor reduces early recurrent ischaemic events compared with aspirin alone.^4^ However, this benefit is time limited and constrained by bleeding risk. For this reason, DAPT is generally restricted to the early post-event phase, commonly for 21 days, before transition to antiplatelet monotherapy.^4^ Combining antiplatelet agents improves protection against early recurrence, but prolonged intensification of platelet inhibition carries a bleeding penalty. Therefore, the next step in secondary prevention is not simply ‘more antiplatelet therapy’, but rather a more selective strategy that targets complementary thrombotic pathways while preserving haemostasis.

In this context, the concept of dual-pathway inhibition becomes particularly relevant. Rather than relying solely on platelet inhibition, dual-pathway prevention combines antiplatelet therapy with selective modulation of the coagulation pathway. The rationale was previously explored in the COMPASS trial.^5^ COMPASS tested low-dose rivaroxaban plus aspirin in patients with chronic coronary artery disease or peripheral artery disease and demonstrated that adding factor (F) Xa inhibition to antiplatelet therapy reduced ischaemic vascular events compared with aspirin alone. In the subgroup of patients with prior stroke, the rate of ischaemic or uncertain stroke was 3.4% per year among aspirin-assigned patients and was reduced by 67% with rivaroxaban plus aspirin.^5^ These data support dual-pathway inhibition in stroke prevention: adding targeted coagulation inhibition to antiplatelet therapy may reduce residual ischaemic risk.

Factor XIa inhibition is an attractive concept because it targets thrombus amplification while preserving haemostasis,^6^ a key requirement in stroke prevention where intracranial bleeding risk is critical.

OCEANIC-STROKE evaluated this concept in a contemporary secondary stroke prevention population. The Phase III trial enrolled 12 327 patients with recent non-cardioembolic ischaemic stroke or high-risk TIA, randomized to receive either asundexian 50 mg daily or placebo alongside standard antiplatelet therapy within 72 h from stroke onset. Most patients (62.6%) were on dual antiplatelet therapy, with 3859 in the asundexian group and 3853 in the placebo group. The observed benefits of asundexian were primarily on top of existing guideline-based stroke prevention strategies. The primary efficacy endpoint was ischaemic stroke, while the primary safety endpoint was International Society of Thrombosis and Haemostasis (ISTH) major bleeding.^2^

The findings demonstrate that asundexian significantly reduced recurrent ischaemic stroke rates compared with placebo, with 6.2% vs. 8.4% events and a cause-specific hazard ratio (HR) of 0.74 [95% confidence interval (CI), 0.65–0.84; *P* < 0.001]. Importantly, the reduction in recurrent ischaemic stroke was achieved without a significant increase in major bleeding, with ISTH major bleeding occurring in 1.9% of patients on asundexian vs. 1.7% on placebo (HR: 1.10, 95% CI, 0.85–1.44), indicating a favourable safety profile. Moreover, OCEANIC-STROKE included patients with different stroke aetiologies including large-artery atherosclerosis, small-vessel disease, stroke of undetermined aetiology, and a small proportion later classified as cardioembolic. The benefit of asundexian was directionally consistent across the aetiologic groups. In large-artery atherosclerosis, ischaemic stroke occurred in 8.1% with asundexian vs. 9.9% with placebo, HR 0.82; 95% CI, 0.68–0.98. In small-vessel occlusion, events occurred in 4.6% vs. 6.7%, HR 0.68; 95% CI, 0.49–0.94. In stroke of undetermined aetiology, a category that may include embolic stroke of undetermined stroke (ESUS)-like patients depending on diagnostic work-up, events occurred in 4.6% vs. 7.4%, HR 0.61; 95% CI, 0.46–0.81. This beneficial effect in ESUS patients is in contrast to previous studies with FXa inhibitors showing no superiority compared to aspirin alone.^7,8^

The above suggests that FXIa inhibition may not be restricted to a single stroke mechanism but may provide protection across different non-cardioembolic pathways.

An open question is the optimal duration of treatment. In OCEANIC-STROKE, the cumulative incidence curves for ischaemic stroke separate early and continue to diverge over follow-up, suggesting that the benefit of asundexian may increase over time rather than being limited to the acute post-event phase. This raises a clinically relevant issue: should FXIa inhibition be used only during a defined high-risk period after stroke/TIA, or continued as longer-term secondary prevention in selected patients?

This is particularly relevant because current DAPT strategies are time limited due to bleeding concerns, whereas OCEANIC-STROKE showed no significant increase in major bleeding with asundexian.

## Primary stroke prevention with factor XIa inhibition in atrial fibrillation: an unsolved issue

In contrast to the efficacy of asundexian observed in OCEANIC-STROKE, outcomes in the higher-risk population with AF were negative.^5^ OCEANIC-AF evaluated whether asundexian 50 mg once daily would be non-inferior to apixaban for the prevention of ischaemic stroke or systemic embolism, superior in reducing bleeding, and capable of providing an overall net clinical benefit in patients with AF at risk of stroke. However, the trial was terminated prematurely as it demonstrated that patients assigned to asundexian experienced a higher incidence of stroke or systemic embolism than those receiving apixaban. The primary outcome occurred in 1.3% of patients assigned to asundexian and in 0.4% of those assigned to apixaban (HR 3.79; 95% CI, 2.46–5.83). Notably, this difference emerged early after randomization and persisted throughout follow-up.

## Mechanistic considerations

These findings highlight a striking difference in thromboembolic outcomes between the two OCEANIC trials. A key question is why the same dose of the same anticoagulant failed in primary stroke prevention in AF but was effective in secondary prevention after non-embolic, largely atherothrombotic, ischaemic stroke?

First, the patient populations differed substantially. Participants in OCEANIC-STROKE had a history of stroke and a high burden of atherosclerotic disease, whereas this was not the case in OCEANIC-AF.^9^ Although patients in OCEANIC-AF were at elevated thromboembolic risk, as reflected by age and CHA_2_DS_2_-VASc score, only a minority had experienced a previous stroke and documented atherosclerotic disease was present in only 33% of participants. One other consideration following OCEANIC-AF was underdosing.^10^ Although asundexian 50 mg once daily was predicted to achieve >90% inhibition of free FXIa,^11^ this level of inhibition may still have been insufficient. Moreover, in AF a difference in anticoagulant potency could emerge; upon comparing asundexian and apixaban in a small substudy of OCEANIC-AF, the latter more effectively reduced thrombin generation *ex vivo*.^12^

Alternative explanations include the possibility that FXI activation is not a dominant driver of thrombus formation in AF-related embolic stroke or that compensatory pathways bypass FXI to sustain thrombin and fibrin generation.^13^ The ongoing LIBREXIA-AF trial will be critical in determining whether milvexian (100 mg twice daily) can reduce the risk of embolic stroke in AF, thereby confirming or refuting the role of FXIa in this heterogeneous condition.^14^ Notably, asundexian and milvexian differ in their *in vitro* anticoagulant profiles, which may translate into differences in clinical efficacy, potentially at the expense of safety.^15^

An important insight from OCEANIC-STROKE is the efficacy advantage of dual-pathway inhibition with asundexian and antiplatelet therapy, without a loss in safety. It will be particularly interesting to further study the combination of asundexian with DAPT in other high-risk atherosclerosis manifestations, as alternative to the COMPASS regimen of low-dose rivaroxaban with aspirin, that is still reluctantly implemented due to its bleeding enhancing risk profile; moreover, it is not registered for use in stroke patients.^16^ On the other hand, if FXIa inhibition is indeed generally weaker than centrally (FXa, thrombin) acting direct oral anticoagulants or vitamin K antagonists, it may limit options in high-risk thrombosis patients, except perhaps for those with an increased bleeding risk.

Potentially, the specific advantage of FXIa inhibition in atherosclerosis may relate to the interplay between coagulation, platelets, and inflammation, revealing some unique properties of FXI(a). Experimental data have shown that thrombin-mediated FXI activation, in a platelet glycoprotein Ibα-dependent manner, contributed to vascular inflammation and endothelial dysfunction, particularly in the context of hypertension.^17^ Such distinct molecular and cellular interactions may create a setting in which FXIa inhibition and antiplatelet therapy act synergistically. Whether these effects are vascular bed specific and/or time dependent remains to be elucidated.

The neutral efficacy signal in the PACIFIC-AMI study with asundexian^18^ and the apparent lack of efficacy of milvexian in the halted LIBREXIA-ACS trial^19^ further highlights the complexity of these mechanisms and suggests that additional differences in vascular territory, disease acuity, and/or thrombogenic drivers are critical.

**Figure 1:**
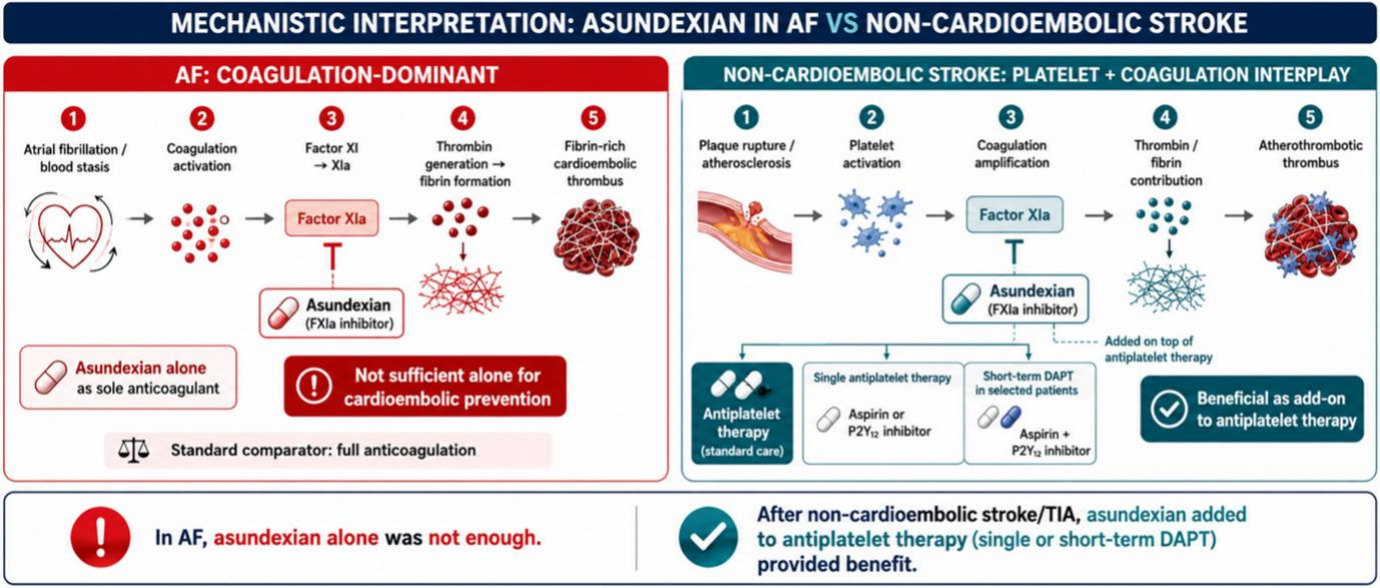
Mechanistic interpretation of asundexian in atrial fibrillation vs. non-cardioembolic ischaemic stroke.

Another consideration in OCEANIC-AF is whether FXIa inhibition may improve recovery from ischaemic stroke by attenuating persistent thrombo-inflammation in the brain. Although a role for platelets in post-stroke immune dysregulation is well documented,^20^ coagulation proteases such as FXIa have not been consistently associated with stroke severity or outcome.^21^ This suggests that the principal benefit of FXIa inhibition following ischaemic stroke may lie in secondary thrombosis prevention rather than modulation of acute brain injury, although an additional endothelial cell stabilizing effect of FXIa inhibition could theoretically contribute to a sustained efficacy benefit, also related to the safety profile (*Figure 1*).^22^

Finally, no clear increase in major adverse cardiovascular events was observed in either OCEANIC trial. Preclinical data showed a protective effect of liver produced FXI against diastolic dysfunction, through activation of the bone morphogenetic protein (BMP)-SMAD1/5 pathway.^23^ Consequently, clinical studies explored the impact of variation in FXI plasma level on heart function, with controversial results.^24^ The OCEANIC-AF study suggests that FXIa inhibition is not associated with overt cardiac harm in populations without pre-existing heart failure.^2^ Nevertheless, caution may be warranted in patients with established heart failure.

Whether interactions with hypertension are clinically relevant remains uncertain, although specific inhibitors of FXI activation have been shown to lower blood pressure in experimental models.^25^ This interaction warrants further investigation, as it may potentially contribute to efficacy outcomes and explain possible differences among ethnic subgroups.^2^

## Conclusions

In conclusion, while OCEANIC-AF did not provide evidence for efficacy of asundexian as sole anticoagulant for primary stroke prevention in AF, OCEANIC-STROKE provides the first positive Phase 3 evidence supporting FXIa inhibition with asundexian as an add-on strategy to antiplatelet therapy for secondary prevention after non-cardioembolic ischaemic stroke or high-risk TIA. The ongoing LIBREXIA-STROKE trial, evaluating milvexian on top of antiplatelet therapy in a similar population, will be essential to determine whether the benefits observed with asundexian reflect a consistent class effect of FXIa inhibition in secondary stroke prevention. The ongoing LIBREXIA-AF study is critical in establishing whether primary stroke prevention in AF is also feasible, by targeting FXIa; time will tell!

## Data Availability

This article is based on publications by others, hence we refer the reader to the original publications for data requests.
